# *N*-[(Methyl­sulfan­yl)meth­yl]benzamide

**DOI:** 10.1107/S1600536812004515

**Published:** 2012-02-10

**Authors:** Muhammad Riaz Khan, Azim Khan, M. Nawaz Tahir, Muhammad Adeel, Saeed Ahmad

**Affiliations:** a Department of Chemistry, Gomal University, Dera Ismail Khan, K.P.K., Pakistan; b University of Sargodha, Department of Physics, Sargodha, Pakistan

## Abstract

In the title compound, C_9_H_11_NOS, the phenyl ring and formamide unit make a dihedral angle of 23.93 (14)°, whereas the (methyl­sulfan­yl)methyl group is oriented at a dihedral angle of 61.31 (8)° with respect to the phenyl ring. There are inter­molecular N—H⋯O hydrogen bonds, forming *C*(4) chains along the [010] direction. These polymeric chains are linked by C—H⋯O hydrogen bonds to form polymeric sheets in the (110) plane.

## Related literature

For crystal structures containing the 1-(methyl­sulfan­yl)methanamine grouping, see: Siddiqui *et al.* (2008 ▶); Noroozi Pesyan *et al.* (2009 ▶). For graph-set notation, see: Bernstein *et al.* (1995 ▶).
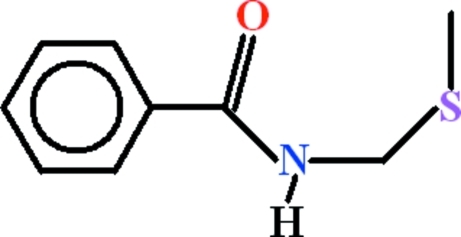


## Experimental

### 

#### Crystal data

C_9_H_11_NOS
*M* *_r_* = 181.25
Orthorhombic, 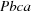

*a* = 9.7841 (4) Å
*b* = 9.2116 (4) Å
*c* = 21.2663 (8) Å
*V* = 1916.67 (14) Å^3^
*Z* = 8
Mo *K*α radiation
μ = 0.29 mm^−1^
*T* = 296 K
0.26 × 0.20 × 0.18 mm

#### Data collection

Bruker Kappa APEXII CCD diffractometer
Absorption correction: multi-scan (*SADABS*; Bruker, 2005 ▶) *T*_min_ = 0.932, *T*_max_ = 0.950
9379 measured reflections
2371 independent reflections
1722 reflections with *I* > 2σ(*I*)
*R*_int_ = 0.022

#### Refinement

*R*[*F* ^2^ > 2σ(*F* ^2^)] = 0.041
*wR*(*F* ^2^) = 0.116
*S* = 1.07
2371 reflections
110 parameters
H-atom parameters constrained
Δρ_max_ = 0.18 e Å^−3^
Δρ_min_ = −0.26 e Å^−3^

### 

Data collection: *APEX2* (Bruker, 2007 ▶); cell refinement: *SAINT* (Bruker, 2007 ▶); data reduction: *SAINT*; program(s) used to solve structure: *SHELXS97* (Sheldrick, 2008 ▶); program(s) used to refine structure: *SHELXL97* (Sheldrick, 2008 ▶); molecular graphics: *ORTEP-3 for Windows* (Farrugia, 1997 ▶) and *PLATON* (Spek, 2009 ▶); software used to prepare material for publication: *WinGX* (Farrugia, 1999 ▶) and *PLATON*.

## Supplementary Material

Crystal structure: contains datablock(s) global, I. DOI: 10.1107/S1600536812004515/wn2467sup1.cif

Structure factors: contains datablock(s) I. DOI: 10.1107/S1600536812004515/wn2467Isup2.hkl

Supplementary material file. DOI: 10.1107/S1600536812004515/wn2467Isup3.cml

Additional supplementary materials:  crystallographic information; 3D view; checkCIF report

## Figures and Tables

**Table 1:** Hydrogen-bond geometry (Å, °)

*D*—H⋯*A*	*D*—H	H⋯*A*	*D*⋯*A*	*D*—H⋯*A*
N1—H1⋯O1^i^	0.86	2.02	2.8438 (17)	160
C8—H8*B*⋯O1^ii^	0.97	2.53	3.434 (2)	154

Symmetry codes: (i) 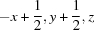; (ii) 
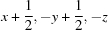
.

**Fig. 1.**
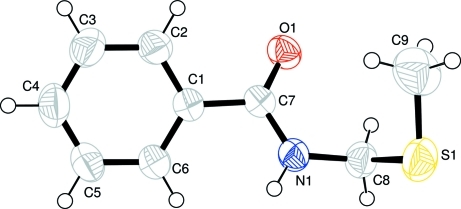
View of the title compound, with displacement ellipsoids drawn at the 50% probability level. H-atoms are shown as small circles of arbitrary radius.

**Fig. 2.**
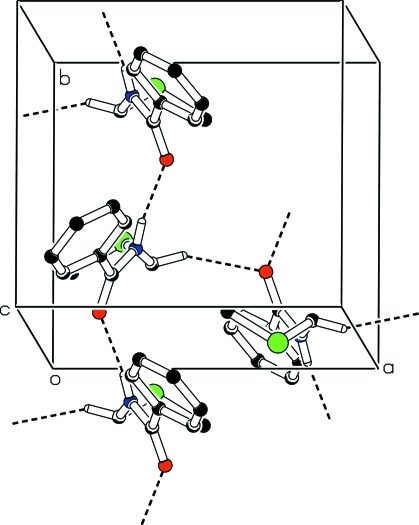
Packing diagram of the title compound (PLATON: Spek, 2009) showing that molecules form one dimensional polymeric chains along [010] and are interlinked. H atoms not involved in hydrogen bonding have been omitted for clarity.

## supplementary crystallographic information

### Comment

The title compound (Fig. 1) was prepared in an attempt to synthesize a different compound from benzamide and phthalic anhydride in dimethyl sulphoxide.

The crystal structures of 2-((methylsulfanyl)methyl)-1,2-benzisothiazol-3(2*H*)-one 1,1-dioxide (Siddiqui *et al.*, 2008) and 5-(2,6-dimethoxyphenoxy)-2-methylsulfanylmethyl-2*H*-tetrazole (Noroozi Pesyan *et al.*, 2009) have been published; these contain the 1-(methylsulfanyl)methanamine grouping.

Let A, B, C denote the planes defined by the phenyl ring (C1–C6), the formamide unit (O1/C7/N1) and the (methylsulfanyl)methane grouping (C8/S1/C9), respectively. The dihedral angles between A/B, A/C and B/C are 23.93 (14)°, 61.31 (8)° and 67.92 (13)°, respectively.

There are intermolecular N—H···O hydrogen bonds (Table 1, Fig. 2), forming C(4) chains (Bernstein *et al.*, 1995) along the [010] direction. These polymeric chains are linked by C—H···O hydrogen bonds (Table 1, Fig. 2) to form polymeric sheets in the (110) plane.

### Experimental

The title compound was prepared by adding a solution of benzamide (0.1 g, 0.078 mmol) in 3 ml of dimethyl sulphoxide (DMSO) to a solution of phthalic anhydride (0.1 g, 0.078 mmol) in DMSO (3 ml). The reaction mixture was heated to 353 K for 6 h. The organic and aqueous layers were separated and the latter was extracted with chloroform (3×15 ml). The organic layer was concentrated *in vacuo* and the residue was purified by chromatography (silica gel, EtOAc/hexane= 1:4). The title compound was obtained as a colorless crystalline solid. Yield = 0.14 g, 70%, m.p = 365 K. Crystallization from a saturated chloroform solution at ambient temperature gave colourless prisms.

### Refinement

The H-atoms were positioned geometrically (N—H = 0.86 Å, C—H = 0.93–0.97 Å) and refined as riding with *U*_iso_(H) = *xU*_eq_(C, N), where *x* = 1.5 for methyl groups and *x* = 1.2 for all other H-atoms.

### Crystal data

**Table:**

C_9_H_11_NOS	*F*(000) = 768
*M**_r_* = 181.25	*D*_x_ = 1.256 Mg m^−^^3^
Orthorhombic, *P**b**c**a*	Mo *K*α radiation, λ = 0.71073 Å
Hall symbol: -P 2ac 2ab	Cell parameters from 1722 reflections
*a* = 9.7841 (4) Å	θ = 1.9–28.3°
*b* = 9.2116 (4) Å	µ = 0.29 mm^−^^1^
*c* = 21.2663 (8) Å	*T* = 296 K
*V* = 1916.67 (14) Å^3^	Prism, colorless
*Z* = 8	0.26 × 0.20 × 0.18 mm

### Data collection

**Table:**

Bruker Kappa APEXII CCD diffractometer	2371 independent reflections
Radiation source: fine-focus sealed tube	1722 reflections with *I* > 2σ(*I*)
Graphite monochromator	*R*_int_ = 0.022
Detector resolution: 7.50 pixels mm^-1^	θ_max_ = 28.3°, θ_min_ = 1.9°
ω scans	*h* = −12→12
Absorption correction: multi-scan (*SADABS*; Bruker, 2005)	*k* = −12→12
*T*_min_ = 0.932, *T*_max_ = 0.950	*l* = −27→28
9379 measured reflections	

### Refinement

**Table:**

Refinement on *F*^2^	Primary atom site location: structure-invariant direct methods
Least-squares matrix: full	Secondary atom site location: difference Fourier map
*R*[*F*^2^ > 2σ(*F*^2^)] = 0.041	Hydrogen site location: inferred from neighbouring sites
*wR*(*F*^2^) = 0.116	H-atom parameters constrained
*S* = 1.07	*w* = 1/[σ^2^(*F*_o_^2^) + (0.0531*P*)^2^ + 0.2845*P*] where *P* = (*F*_o_^2^ + 2*F*_c_^2^)/3
2371 reflections	(Δ/σ)_max_ < 0.001
110 parameters	Δρ_max_ = 0.18 e Å^−^^3^
0 restraints	Δρ_min_ = −0.26 e Å^−^^3^

### Special details

**Table:**

Geometry. Bond distances, angles *etc*. have been calculated using the rounded fractional coordinates. All su's are estimated from the variances of the (full) variance-covariance matrix. The cell e.s.d.'s are taken into account in the estimation of distances, angles and torsion angles
Refinement. Refinement of *F*^2^ against ALL reflections. The weighted *R*-factor *wR* and goodness of fit *S* are based on *F*^2^, conventional *R*-factors *R* are based on *F*, with *F* set to zero for negative *F*^2^. The threshold expression of *F*^2^ > σ(*F*^2^) is used only for calculating *R*-factors(gt) *etc*. and is not relevant to the choice of reflections for refinement. *R*-factors based on *F*^2^ are statistically about twice as large as those based on *F*, and *R*- factors based on ALL data will be even larger.

### Fractional atomic coordinates and isotropic or equivalent isotropic displacement parameters (Å^2^)

**Table:**

	*x*	*y*	*z*	*U*_iso_*/*U*_eq_	
S1	0.20155 (6)	0.43391 (6)	−0.09248 (2)	0.0717 (2)	
O1	0.14747 (12)	0.17375 (11)	0.05060 (5)	0.0537 (4)	
N1	0.25653 (14)	0.38548 (15)	0.03218 (5)	0.0493 (4)	
C1	0.15800 (14)	0.34302 (14)	0.13470 (7)	0.0427 (4)	
C2	0.04865 (18)	0.28086 (18)	0.16623 (8)	0.0562 (5)	
C3	0.0203 (2)	0.3207 (2)	0.22745 (9)	0.0710 (7)	
C4	0.1003 (2)	0.4214 (2)	0.25775 (9)	0.0709 (7)	
C5	0.2082 (2)	0.4838 (2)	0.22691 (8)	0.0648 (6)	
C6	0.23778 (18)	0.44502 (17)	0.16551 (7)	0.0525 (5)	
C7	0.18659 (14)	0.29393 (15)	0.06896 (7)	0.0424 (4)	
C8	0.29981 (18)	0.3490 (2)	−0.03071 (7)	0.0559 (6)	
C9	0.0467 (2)	0.3318 (3)	−0.08796 (11)	0.0905 (9)	
H1	0.27659	0.46996	0.04670	0.0592*	
H2	−0.00569	0.21226	0.14612	0.0674*	
H3	−0.05341	0.27902	0.24832	0.0852*	
H4	0.08116	0.44708	0.29912	0.0851*	
H5	0.26188	0.55253	0.24731	0.0778*	
H6	0.31140	0.48753	0.14487	0.0630*	
H8A	0.29434	0.24449	−0.03582	0.0670*	
H8B	0.39487	0.37666	−0.03557	0.0670*	
H9A	0.00311	0.34980	−0.04829	0.1358*	
H9B	0.06706	0.23019	−0.09169	0.1358*	
H9C	−0.01321	0.36045	−0.12147	0.1358*	

### Atomic displacement parameters (Å^2^)

**Table:**

	*U*^11^	*U*^22^	*U*^33^	*U*^12^	*U*^13^	*U*^23^
S1	0.0957 (4)	0.0699 (4)	0.0496 (3)	−0.0010 (3)	−0.0003 (2)	0.0110 (2)
O1	0.0662 (7)	0.0395 (6)	0.0553 (6)	−0.0005 (5)	−0.0022 (5)	−0.0059 (5)
N1	0.0605 (8)	0.0426 (7)	0.0448 (7)	−0.0021 (6)	0.0027 (6)	−0.0046 (5)
C1	0.0465 (8)	0.0375 (7)	0.0440 (7)	0.0072 (6)	−0.0033 (6)	0.0001 (6)
C2	0.0588 (10)	0.0498 (9)	0.0600 (9)	−0.0024 (7)	0.0056 (8)	−0.0074 (7)
C3	0.0765 (13)	0.0686 (11)	0.0679 (12)	−0.0042 (10)	0.0239 (9)	−0.0067 (9)
C4	0.0883 (14)	0.0736 (12)	0.0509 (10)	0.0057 (11)	0.0117 (9)	−0.0129 (9)
C5	0.0733 (12)	0.0685 (11)	0.0526 (10)	−0.0033 (9)	−0.0067 (8)	−0.0134 (9)
C6	0.0541 (9)	0.0566 (9)	0.0468 (8)	−0.0028 (7)	−0.0045 (7)	−0.0024 (7)
C7	0.0445 (8)	0.0372 (7)	0.0454 (8)	0.0059 (6)	−0.0057 (6)	−0.0001 (6)
C8	0.0568 (10)	0.0632 (10)	0.0476 (9)	0.0011 (8)	0.0064 (7)	−0.0031 (8)
C9	0.0826 (15)	0.0997 (17)	0.0893 (15)	0.0017 (13)	−0.0291 (12)	0.0092 (12)

### Geometric parameters (Å, º)

**Table:**

S1—C8	1.8060 (17)	C5—C6	1.384 (2)
S1—C9	1.786 (2)	C2—H2	0.9300
O1—C7	1.2347 (17)	C3—H3	0.9300
N1—C7	1.3384 (19)	C4—H4	0.9300
N1—C8	1.4426 (19)	C5—H5	0.9300
N1—H1	0.8600	C6—H6	0.9300
C1—C6	1.386 (2)	C8—H8A	0.9700
C1—C7	1.496 (2)	C8—H8B	0.9700
C1—C2	1.386 (2)	C9—H9A	0.9600
C2—C3	1.381 (3)	C9—H9B	0.9600
C3—C4	1.374 (3)	C9—H9C	0.9600
C4—C5	1.369 (3)		
			
C8—S1—C9	100.62 (10)	C4—C3—H3	120.00
C7—N1—C8	123.03 (14)	C3—C4—H4	120.00
C8—N1—H1	118.00	C5—C4—H4	120.00
C7—N1—H1	118.00	C4—C5—H5	120.00
C2—C1—C7	118.13 (13)	C6—C5—H5	120.00
C2—C1—C6	119.07 (14)	C1—C6—H6	120.00
C6—C1—C7	122.78 (13)	C5—C6—H6	120.00
C1—C2—C3	120.08 (16)	S1—C8—H8A	109.00
C2—C3—C4	120.47 (18)	S1—C8—H8B	109.00
C3—C4—C5	119.86 (18)	N1—C8—H8A	109.00
C4—C5—C6	120.31 (17)	N1—C8—H8B	109.00
C1—C6—C5	120.20 (16)	H8A—C8—H8B	108.00
O1—C7—N1	122.59 (14)	S1—C9—H9A	109.00
O1—C7—C1	120.58 (13)	S1—C9—H9B	109.00
N1—C7—C1	116.83 (12)	S1—C9—H9C	109.00
S1—C8—N1	114.66 (12)	H9A—C9—H9B	109.00
C1—C2—H2	120.00	H9A—C9—H9C	109.00
C3—C2—H2	120.00	H9B—C9—H9C	109.00
C2—C3—H3	120.00		
			
C9—S1—C8—N1	−73.54 (15)	C2—C1—C7—O1	−23.5 (2)
C8—N1—C7—O1	−3.9 (2)	C2—C1—C7—N1	157.05 (14)
C8—N1—C7—C1	175.55 (13)	C6—C1—C7—O1	155.07 (15)
C7—N1—C8—S1	105.09 (16)	C6—C1—C7—N1	−24.4 (2)
C6—C1—C2—C3	0.0 (2)	C1—C2—C3—C4	−0.4 (3)
C7—C1—C2—C3	178.63 (15)	C2—C3—C4—C5	0.6 (3)
C2—C1—C6—C5	0.0 (2)	C3—C4—C5—C6	−0.5 (3)
C7—C1—C6—C5	−178.51 (15)	C4—C5—C6—C1	0.2 (3)

### Hydrogen-bond geometry (Å, º)

**Table:**

*D*—H···*A*	*D*—H	H···*A*	*D*···*A*	*D*—H···*A*
N1—H1···O1^i^	0.86	2.02	2.8438 (17)	160
C8—H8*B*···O1^ii^	0.97	2.53	3.434 (2)	154

Symmetry codes: (i) −*x*+1/2, *y*+1/2, *z*; (ii) *x*+1/2, −*y*+1/2, −*z*.
